# Nasopharyngeal Bacterial Interactions in Children

**DOI:** 10.3201/eid2002.131701

**Published:** 2014-02

**Authors:** Anthony Almudevar

**Affiliations:** University of Rochester Medical Center, Rochester, New York, USA

**Keywords:** bacterial colonization, acute otitis media, Haemophilus influenzae, Streptococcus pneumoniae, bacteria, respiratory infections

**In Response:** The point made by Suzuki et al. (*1*) is an interesting one, but it is not directly relevant to our conclusions (*2*). To summarize, given bacterium 1 and bacterium 2, suppose OR[pop] is the odds ratio (OR) between bacterium 1 and bacterium 2 in the population. If r[c] and r[n] are the risk of enrollment among colonization-positive and colonization-negative children, respectively, then OR[case] = (r[n]/r[c])OR[pop], so that OR[case] <1 can be attributed to a higher risk among colonized children.

The underlying assumption of the analysis is that enrollment risk is constant for both bacteria (alone or in combination). In fact, differential risk does exist, and it cannot be separated from the issue of the relative aggressiveness of the bacterium. This can be seen by considering the ORs in the Table.

When we compared the ORs between bacteria pairs for all subjects and children with acute otitis media (AOM), we found a large decrease for pairs involving nontypeable *Haemophilus influenzae* (NTHi), in contrast to the remaining pair, *Streptococcus pneumoniae*/ *Moraxella catarrhailis* (*Spn*/*Mcat*). We do not believe that these values are explainable by the effect described by Suzuki et al., especially when (as is done in that analysis) we assume a colonization-positive risk of enrollment r[c] that is independent of bacterium distribution. We also point out that these estimates are instructive, but not sufficient, because each pairwise comparison may depend on interactions with the third bacterium. We therefore used a statistical model (*2**,**3*) that permits the isolation of third-order effects by modeling co-occurrence rates of 2 bacteria while controlling for a third. This allowed us to reach our conclusion, which is primarily concerned with the specific role played by NTHi, and follows from the existence of a pattern in the reported ORs, rather than the absolute value of any single OR.

It is also instructive to examine the colonization rates for AOM versus number of AOM events (nAOM) subjects: p(*Spn-*nAOM) = 0.30; p(*Spn*-AOM) = 0.53; p(NTHi-nAOM) = 0.12; p(NTHi-AOM) = 0.48; p(*Mcat* -nAOM) = 0.36; p(*Mcat*-AOM) = 0.43. As we would expect, colonization rates of each bacterium are higher for AOM patients. What is of interest is that the colonization distribution is different for children with AOM, which suggests that NTHi is more aggressive than other bacteria in some sense, and this effect is made more precise by the statistical model we used. The essential point is that the issue of competitive association cannot be isolated from differential enrollment risks, which is what our analysis reports.

**Table:** Comparison of the odds ratios between bacterium pairs for all subjects and children with acute otitis media*

Bacteria pair	Odds ratios, p values	
	All subjects	Acute otitis media
NTHi/*Mcat*	0.94, p = 0.6935	0.47, p = 0.0006
NTHi/*Spn*	1.58, p = 0.0004	0.50, p = 0.001
*Spn*/*Mcat*	1.71, p<0.0001	1.53, p = 0.05

*NTHi, nontypeable *Haemophilus influenzae*; Mcat, *Moraxella catarrhailis*; Spn, *Streptococcus pneumoniae*.

Source: (*2*)
